# TIP60-miR-22 axis as a prognostic marker of breast cancer progression

**DOI:** 10.18632/oncotarget.5636

**Published:** 2015-10-19

**Authors:** Amit Kumar Pandey, Yanzhou Zhang, Siting Zhang, Ying Li, Greg Tucker-Kellogg, Henry Yang, Sudhakar Jha

**Affiliations:** ^1^ Cancer Science Institute of Singapore, Yong Loo Lin School of Medicine, National University of Singapore, Singapore; ^2^ Department of Biochemistry, Yong Loo Lin School of Medicine, National University of Singapore, Singapore; ^3^ Department of Biological Sciences, Faculty of Science, National University of Singapore, Singapore

**Keywords:** cancer, TIP60, KAT5, EMT, miR-22

## Abstract

MicroRNAs (miRNAs) are 22- to 24-nucleotide, small, non-coding RNAs that bind to the 3′UTR of target genes to control gene expression. Consequently, their dysregulation contributes to many diseases, including diabetes and cancer. miR-22 is up-regulated in numerous metastatic cancers and recent studies have suggested a role for miR-22 in promoting stemness and metastasis. TIP60 is a lysine acetyl-transferase reported to be down-regulated in cancer but the molecular mechanism of this reduction is still unclear. In this study, we identify TIP60 as a target of miR-22. We show a negative correlation in the expression of TIP60 and miR-22 in breast cancer patients, and show that low levels of TIP60 and high levels of miR-22 are associated with poor overall survival. Furthermore, pathway analysis using high miR-22/low TIP60 and low miR-22/high TIP60 breast cancer patient datasets suggests association of TIP60/miR-22 with epithelial-mesenchymal transition (EMT), a key alteration in progression of cancer cells. We show that blocking endogenous miR-22 can restore TIP60 levels, which in turn decreases the migration and invasion capacity of metastatic breast cancer cell line. These results provide mechanistic insight into TIP60 regulation and evidence for the utility of the combination of TIP60 and miR-22 as prognostic indicator of breast cancer progression.

## INTRODUCTION

Breast cancer is one of the most common and significant malignant diseases in women worldwide [1]. Although improvements in detection and treatment have decreased breast cancer mortality in recent years, the stage of detection and ability of cancer cells to metastasize to distant organs have been the major challenges in the successful prevention of and therapy, for this deadly disease. Cancer metastasis is a complex, multi-step process and is driven, promoted, and modulated by aberrantly deregulated cellular signals.

MicroRNAs (miRNAs) are a family of small non-protein-coding RNA molecules of approximately 22–24 nucleotides (nt) that function as key regulators of gene expression at the post-transcriptional level [2]. Since their initial discovery in *Caenorhabditis elegans* [3], thousands of microRNAs have been annotated and currently 2588, 765 and 1915 mature miRNA sequences in human, rat and mouse, respectively, have been catalogued in the microRNA registry (http://www.mirbase.org, V 21 June, 2014). miRNA dysregulation has been shown to contribute to the etiology of multiple diseases, including cancer, where miRNAs can act as either oncogenes or tumor suppressors [4–8]. Indeed, emerging evidence demonstrates that aberrant miRNA expression is linked to breast cancer progression [9, 10].

TIP60 (lysine acetyl-transferase) is part of a conserved multisubunit complex, NuA4, which is recruited by many transcription factors to their target promoters, where it acetylates histones and is involved in transcriptional regulation. TIP60 has been shown to play an important role in many processes such as cellular signaling, DNA damage repair and apoptosis [11, 12], as well as cell cycle and checkpoint control [13]. Involvement of TIP60 in these various processes implies that its expression, stability and localization are regulated in the cell by various mechanisms.

In the current study, we show the first evidence of a non-coding RNA as regulator of TIP60 expression. We find the expression of miR-22 and TIP60 to be negatively correlated in invasive breast cancer tissues and breast cancer cell lines. Furthermore, we identified TIP60 as a miR-22 target and show that, by targeting TIP60, miR-22 stimulates the expression of epithelial-mesenchymal transition (EMT) genes. Using various cell culture models, we find miR-22 expression results in increased cell migration and invasion. Our data suggest that TIP60 and miR-22 could act as prognostic markers in breast cancer disease progression and that targeting the TIP60–miR-22 axis could lead to an effective therapeutic strategy for metastatic breast cancer.

## RESULTS

### TIP60 is a direct target of miR-22

**Figure 1:**
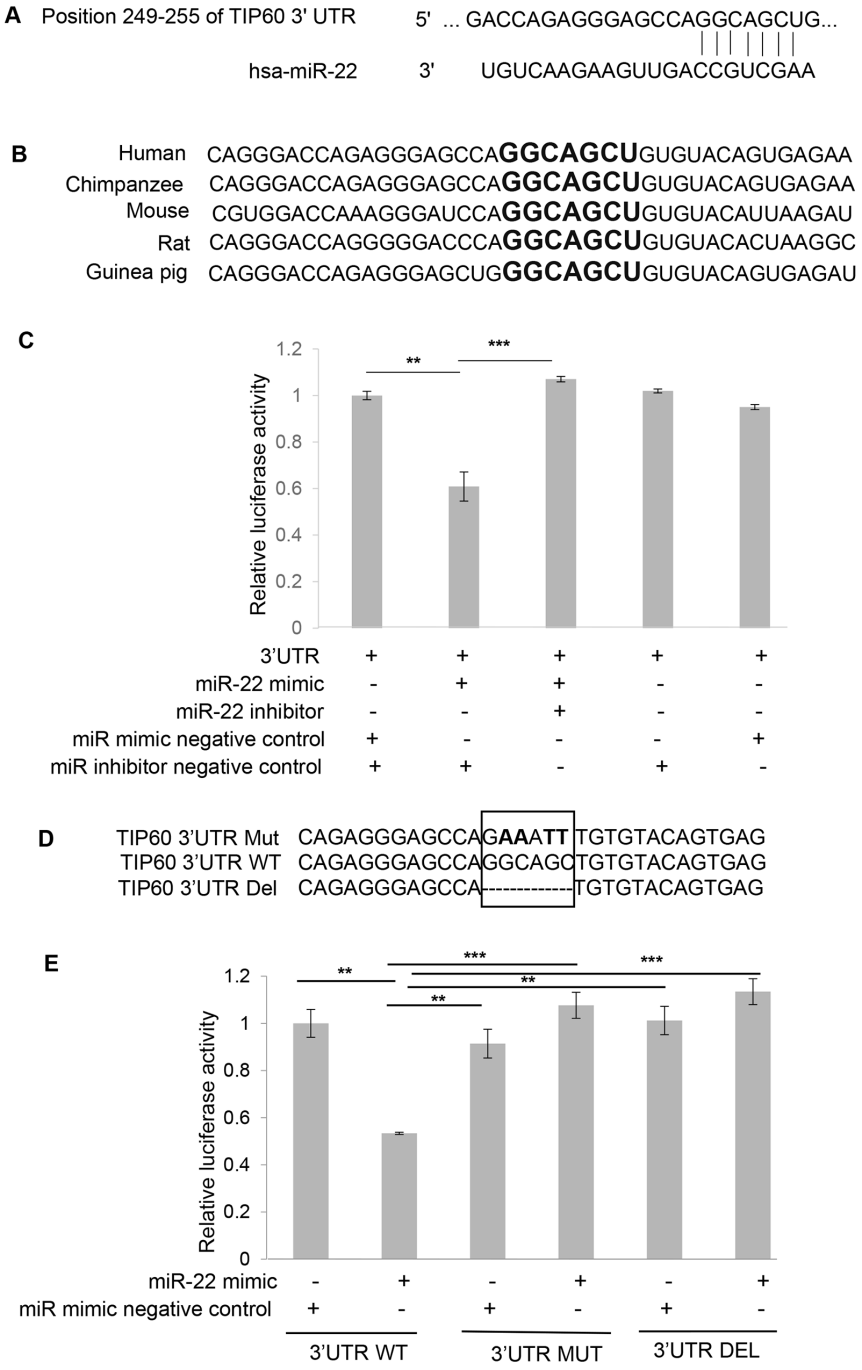
miR-22 binding site at the TIP60 3′UTR **A.** Putative target binding site for miR-22 at the 3′UTR of the TIP60 gene, as predicted by Targetscan (http://www.targetscan.org/). **B.** The target site is highly conserved across the various indicated species. Highlighted nucleotides (in bold) indicate the putative miR-22 binding site. **C.** The miR-22 binding site on TIP60 3′UTR was confirmed by luciferase activity in MCF7 cells after co-transfection of pmirGLO-TIP60 3′-UTR plasmid with the indicated miRs (50 nM). **D.** Mutation of the miR-22 binding site in the 3′UTR of TIP60. **E.** MCF7 cells were co-transfected with a wild-type pmirGLO-TIP60 3′-UTR luciferase construct, or a construct containing a mutation in the predicted miR-22 binding site or construct having binding site deleted with either the miR-22 mimic or the negative control mimic. Luciferase expression was normalized to Renilla luciferase and the data is depicted as the mean ± SEM. The figure summarizes data from three independent experiments performed in triplicate. Analysis was performed using an unpaired two-tailed student's *t*-test. Significance is represented as ***P < 0.001; **P < 0.01.

TIP60 is known to be down-regulated in multiple cancers [14, 15]. Whereas we and others have identified TIP60 to be destabilized by viral oncogenes [16–19], other potential mechanisms of its downregulation are unknown. In order to investigate whether TIP60 expression could be regulated by miRNAs, we performed an *in silico* analysis using the Targetscan database (http://www.targetscan.org/) to identify putative miRNA seed-matching sequences in TIP60. We found one putative target binding site for miR-22 at the position 249–255 nt in the 3′UTR of TIP60 (Figure 1A). This identified seed sequence was also conserved among different species of TIP60, indicating the likely functional importance of this motif (Figure 1B). To further validate TIP60 as a target of miR-22, we cloned the 3′UTR of TIP60 into the pmirGLO dual-luciferase vector, and transiently co-transfected pmirGLO-TIP60 WT 3′UTR into MCF7 cells along with a miRNA mimic negative control (that does not target any known mRNA within the human transcriptome) or a miR-22 mimic either alone or in combination with miR inhibitor negative control. A miR-22 hairpin inhibitor was also transfected and used to show specificity of miR-22 for TIP60. After 48 h of transfection, cells were lysed and the protein was analyzed for luciferase activity. We measured a 40% reduction in the luciferase activity of pmirGLO-TIP60 WT 3′UTR with miR-22 mimic overexpression (Figure 1C), and this reduction could be rescued upon the co-transfection with the miR-22 hairpin inhibitor, suggesting specificity of this regulation (Figure 1C). In addition, we did not observe any difference in luciferase activity when pmirGLO-TIP60 WT 3′UTR was transfected with either miR mimic negative control or with miR inhibitor negative control alone, suggesting target specificity. To further demonstrate that the decrease in luciferase activity is due to miR-22 binding to the seed sequence in the 3′ UTR of TIP60, we generated two 3′UTR mutant constructs: the first comprised point mutations in the miR-22 binding sites of TIP60 (pmirGLO-TIP60 Mut 3′UTR); in the second, we deleted the miR-22 seed sequence at the TIP60 3′UTR using site-directed mutagenesis (pmirGLO-TIP60 Del 3′UTR). Clones were confirmed by sequencing (Figure 1D). These mutants were then co-transfected along with the miR mimic negative control or miR-22 mimic. We observed no repression in luciferase activity after mutating or deleting the binding site (Figure 1E), suggesting that miR-22 directly interacts with the TIP60 3′UTR and targets TIP60.

### miR-22 and TIP60 expression is negatively co-related

**Figure 2:**
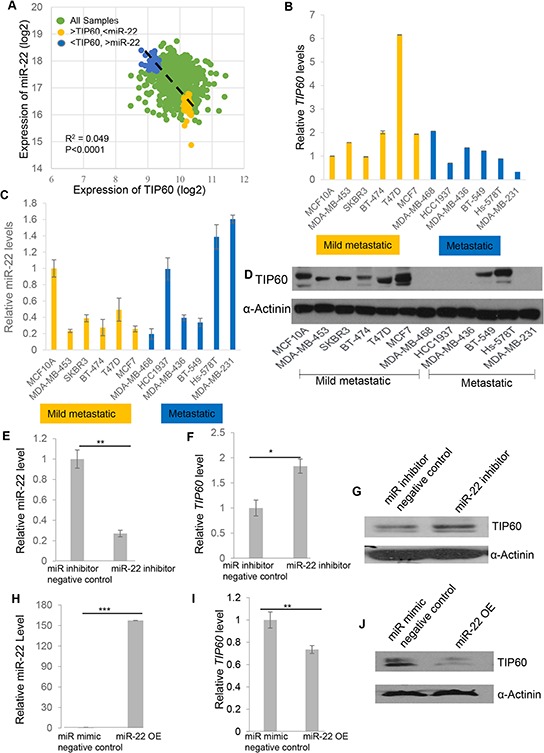
Negative correlation of TIP60 and miR-22 mRNA and protein levels in breast cancer patients and cell lines **A.** Negative correlation between miR-22 and TIP60 mRNA levels for each individual sample from the TCGA dataset. **B, C.** The expression of *TIP60* mRNA and miR-22 mRNA was detected by QRT-PCR in a panel of 12 breast cancer cell lines. Mild metastatic and metastatic cell lines are shown in yellow and blue colors, respectively. **D.** Western blot showing the expression of TIP60 in a panel of 12 breast cancer cell lines **E.** mRNA levels of miR-22 were decreased on miR-22 inhibition in MDA-MB-231 cells when transfected with miR-22 inhibitor for 48 h (50 nM). **F–G.** mRNA and protein levels of TIP60 were increased following inhibition of miR-22 in MDA-MB-231 cells when transfected with miR-22 inhibitor for 48 h (50 nM). **H.** Relative miR-22 levels were determined by QRT-PCR on miR-22 mimic overexpression (miR-22OE, 50 nM) in MCF7 cells. **I–J.** mRNA and protein levels of TIP60 were decreased following miR-22 mimic overexpression (50 nM) in MCF7 cells. The figure illustrates data from three independent experiments performed in triplicate. Significance is represented as *P < 0.05; **P < 0.01; ***P < 0.001.

Having identified a miR-22 binding site in the 3′UTR of TIP60, we next sought to understand the physiological relevance of this regulation. We decided to focus on breast cancer, as a recent study by Song et al. [20] implicated the role of miR-22 in breast cancer. To this end, we analyzed the expression of TIP60 and miR-22 in a breast cancer dataset from The Cancer Genome Atlas (TCGA) database and found a small but significant negative correlation between TIP60 and miR-22 expression (Figure 2A). To investigate the potential biological significance of this negative correlation, we sought to identify a cell culture model that also showed a negative correlation between TIP60 and miR-22. For this, we analyzed the expression of miR-22 and TIP60 (mRNA and protein) in 12 breast cancer cell lines on the basis of their EMT score as described by Tan et.al. [21]. Interestingly, mesenchymal cell lines such as MDA-MB-231, Hs578T and MDA-MB-468 and epithelial cell line such as MCF-7 and T47D showed a negative correlation between miR-22 and TIP60 mRNA expression (Figure 2B and 2C), with high miR-22 and low TIP60 expression in the highly metastatic MDA-MB-231 cell line and Hs578T cell line, but high TIP60 and low miR-22 expression in the MCF7, T47D mild metastatic cells and MDA-MB-468 basal, triple-negative cells. A similar expression profile of TIP60 was also observed at the protein level (Figure 2D).

To further investigate whether miR-22 affects endogenous TIP60 expression, we focused on 2 of the 12 breast cancer cell lines: MCF7, an epithelial cell line that is mildly metastatic and has low miR-22 and high TIP60 expression and MDA-MB-231 which is a mesenchymal cell line, highly metastatic and has high miR-22 and low TIP60 expression. We then transfected the miR‐22 mimic or miR mimic negative control and compared the level of TIP60 protein in MCF7 cells under these two conditions. Similarly, the MDA-MB-231 cell line was transfected with the miR-22 inhibitor or with a miR inhibitor negative control to examine endogenous changes in TIP60. We found that TIP60 expression was increased in MDA-MB-231 cells (Figure 2F–2G) and reduced in MCF7 cells (Figure 2I–2J) after inhibition (Figure 2E) or overexpression of miR-22 mimic (Figure 2H), respectively both at mRNA and protein level. These data indicate that miR-22 down-regulates endogenous TIP60 expression.

### miR-22 regulates EMT genes by repressing TIP60

**Figure 3:**
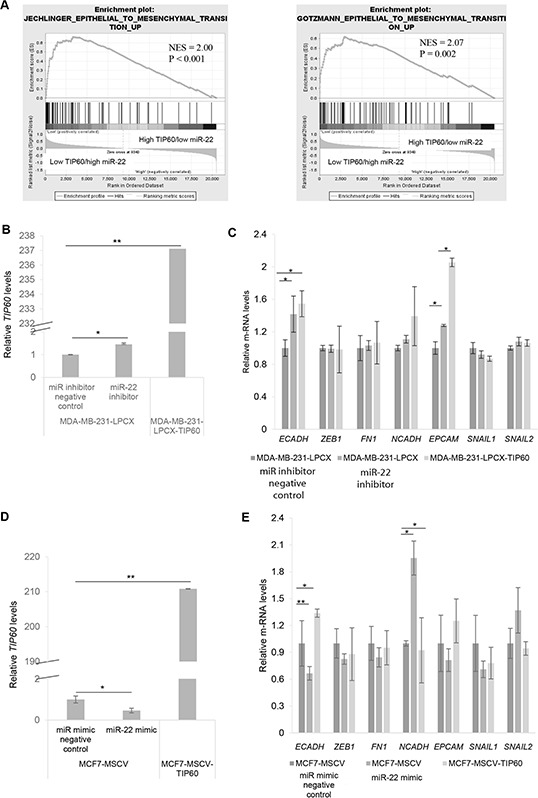
Expression levels of TIP60 and miR-22 suggest an epithelial-mesenchymal transition (EMT) **A.** Gene-Set Enrichment Analysis (GSEA) shows enrichment of factors linked to EMT in the samples with high miR-22 and low TIP60. **B.** The expression of TIP60 mRNA was detected by QRT-PCR in cells transfected with either miR inhibitor negative control or miR-22 inhibitor and also MDA-MB-231-LPCX-TIP60 stable cell line. **C.** QRT-PCR showing mRNA expression of EMT markers in cells transfected with either miR inhibitor negative control or miR-22 inhibitor and also MDA-MB-231-LPCX-TIP60 stable cell line. **D.** The expression of TIP60 mRNA was detected by QRT-PCR in cells transfected with either miR mimic negative control or miR-22 mimic overexpression and also MCF7-MSCV-TIP60 stable cell line. **E.** QRT-PCR showing mRNA expression of EMT markers in cells transfected with either miR mimic negative control or miR-22 mimic overexpression and also MCF7-MSCV-TIP60 stable cell line. The figure represents data from three independent experiments performed in triplicate. Significance is represented as *P < 0.05; **P < 0.01.

**Figure 4:**
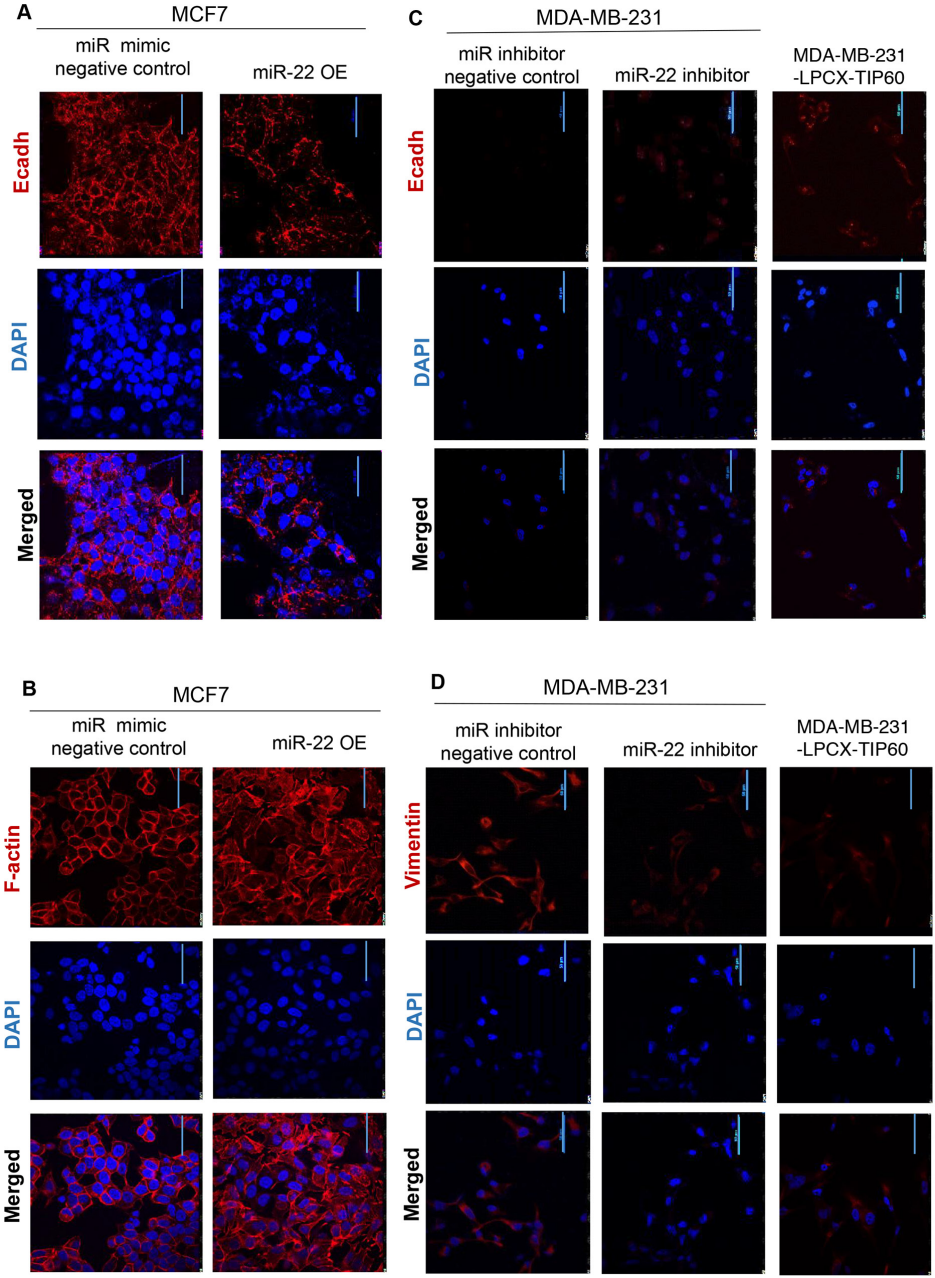
Immuno-fluorescence showing phenotypic alterations **A–B.** Representative immuno-fluorescence images of E-cadherin and F-actin (red) are shown for MCF7 cells transfected with either miR mimic negative control or miR-22 mimic. Nuclei are stained with DAPI (blue). **C–D.** Representative immuno-fluorescence images of E-cadherin and Vimentin (red) are shown for MDA-MB-231 cells transfected with either miR-22 inhibitor negative control or miR-22 inhibitor or MDA-MB-231-LPCX-TIP60 stable cell line. Nuclei are stained with DAPI (blue). Immuno-fluorescence images were taken at 60X magnification with a nikon confocal microscope.

To determine the downstream effects of TIP60 regulation by miR-22, we performed Gene-Set Enrichment Analysis (GSEA) using msigdb.v4 (http://www.broad.mit.edu/gsea/) and compared 28 TCGA samples with high TIP60/low miR-22 expression with 28 samples with low TIP60/high miR-22 expression. Our GSEA analysis revealed enrichment of the epithelial-mesenchymal transition (EMT) pathway (Figure 3A). Since EMT is related to cellular migration and invasion, we sought to determine the effect of miR-22 on various EMT markers. We ablated miR-22 activity by transfecting MDA-MB-231 cells with the miR-22 inhibitor or overexpressed the miR-22 mimic in MCF7 cells. Aside from increased TIP60 levels (Figure 3B), we found that miR-22 inhibition in MDA-MB-231 cells resulted in increased *E-cadherin* levels, an epithelial marker that is lost upon execution of the EMT program (Figure 3C). Similar increase in *E-cadherin* was also found in MDA-MB-231-LPCX-TIP60 stable cell line. In comparison, MCF7 cells showed decreased TIP60 (Figure 3D) and *E-cadherin* levels (Figure 3E) and increased *N-cadherin* (mesenchymal marker) levels in the presence of miR-22 mimic (Figure 3E) and this effect was rescued on overexpressing TIP60 in MCF7 cell line. Further, we did not observe any changes in other EMT markers. These data suggest that miR-22 induces EMT like phenotype and this is associated with a change in the expression of TIP60. The phenotypic alterations induced by miR-22 mimic overexpression in MCF7 cells is observed by immuno-fluorescence staining of the E-cadherin. MCF7 cells treated with miR mimic negative control showed expression of E-cadherin (Figure 4A, mimic negative control) and this was reduced in miR-22 mimic overexpressed MCF7 cells (Figure 4A, miR-22 OE). We further examined the status of F-actin in the cells by phalloidin staining, since actin reorganization occurs during the EMT process [22]. In contrast to miR mimic negative control treated cells (Figure 4B, mimic negative control), overexpression of miR-22 mimic significantly induced actin fiber formation, typical of EMT (Figure 4B, miR-22 OE). These results indicated that the epithelial property of the cells might be lost when miR-22 mimic is overexpressed. We next examined whether miR-22 inhibition in MDA-MB-231 cell line shows the opposite effect. Indeed, inhibition of miR-22 activity in MDA-MB-231 cells showed increased E-cadherin expression (Figure 4C, miR-22 inhibitor) and decreased Vimentin expression (Figure 4D, miR-22 inhibitor), which suggested the reversal of the EMT process and this effect was also observed in TIP60-overexpression MDA-MB-231 cell line (Figure 4C, 4D).

### miR-22 inhibition suppresses cell migration and invasion by regulating TIP60 levels

**Figure 5:**
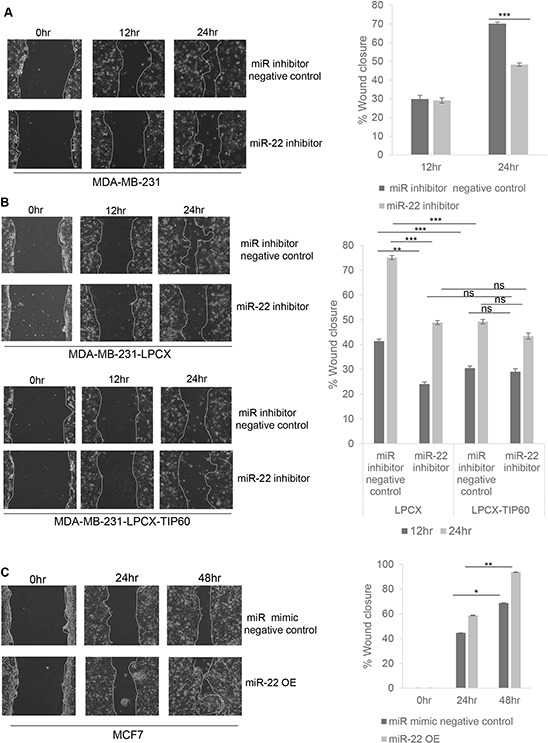
miR-22 increases cell migration by targeting expression of TIP60 MDA-MB-231 cells **A.** and MDA-MB-231-LPCX and MDA-MB-231-LPCX-TIP60 cells **B.** were treated with or without the miR-22 inhibitor for 24 h and analyzed by wound-healing assays 48 h after transfection using live-cell imaging (Nikon). The solid white line highlights the wound edge at 0 h and 24 h. **C.** MCF7 cells were transfected with miR-22 mimic and wound closure was analyzed as for (A) and (B) Data compiled from three independent experiments in triplicate, and images from one representative experiment are shown. Decreases in the gap area between the migrating cells from the opposite wound edge were quantified by measuring the distance (by scale) at three random points in the image. This quantification is represented in the figure. Data are the mean ± SEM with significance measured using unpaired two-tailed student's *t*-test. Significance is represented as *P < 0.05; ** *P* < 0.01; ***P < 0.001.

Two key features of EMT are the ability of cells to migrate and invade. In order to investigate the role of miR-22 in these processes, we performed wound-healing and cell invasion assays. For this, MDA-MB-231 cells were transfected with miR-22 hairpin inhibitor or control hairpin inhibitor for 48 h and cells were serum starved for the next 12 h. We found that inhibiting miR-22 caused a significant decrease in the rate of wound closure in the MDA-MB-231 cell cultures at 12 h and 24 h as compared to that of control cells (Figure 5A). To further demonstrate that miR-22 increased cell migration through TIP60, we performed the wound-healing assay in MDA-MB-231 cell line stably overexpressing TIP60 without miR-22 target sequence. Interestingly, overexpression of TIP60 decreased cell migration and we did not observe any effect on migration in the presence of miR-22 inhibitor (Figure 5B). On the other hand, when miR-22 mimic was overexpressed in MCF7 cells, we observed a significant increase in cell migration as compared to the control cells (transfected with miR mimic negative control; Figure 5C). These findings suggest that repression of TIP60 by miR-22 increases cell migration and this can be reverted by ablating activity of miR-22 or through the overexpression of a TIP60 that lacks miR-22 binding site. Thus, miR-22 stimulates cell migration by targeting TIP60.

**Figure 6:**
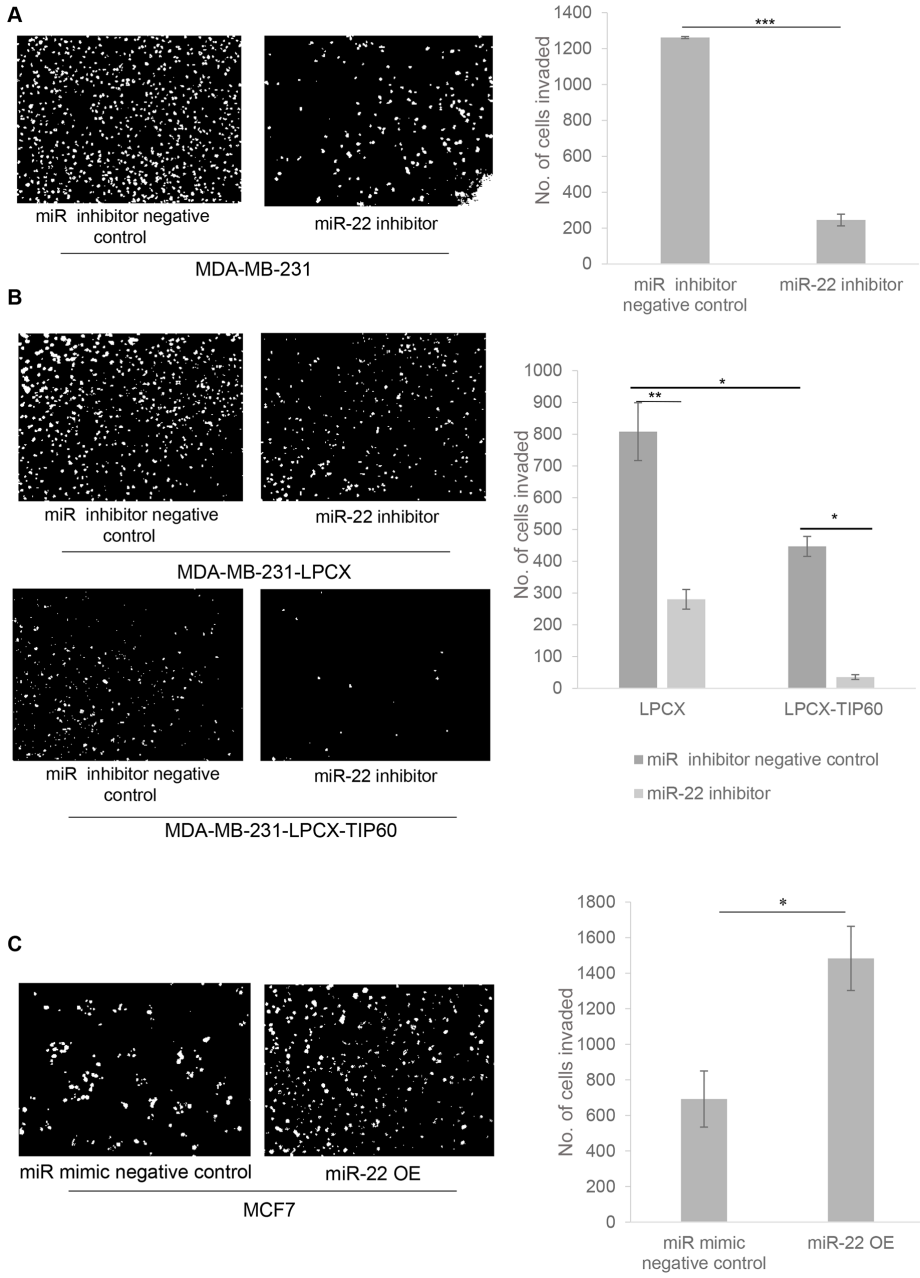
miR-22 inhibition results in decreased cell invasion **A.** MDA-MB-231 cells were treated with or without miR-22 inhibitor and analyzed for their ability to invade into Matrigel transwell 48 h after transfection. **B.** MDA-MB-231-LPCX and MDA-MB-231-LPCX-TIP60 cells were treated with or without miR-22 inhibitor and analyzed using an invasion assay 48 h after transfection. miR-22 decreased MDA-MB-231-LPCX and MDA-MB-231-LPCX-TIP60 cell invasion, with a similar effect also seen in the TIP60 stable cell lines, as compared with vector alone. **C.** MCF7 cells were transfected with miR-22 mimic and wound closure was analyzed 48 h after transfection. miR-22 mimic overexpression increases cell invasion. Data compiled from three independent experiments in triplicate, and images from one representative experiment are shown. Data are the mean ± SEM and significance was determined using an unpaired two-tailed student's *t*-test. Significance is represented as *P < 0.05; **P < 0.01; ***P < 0.001.

To assess invasion, we transfected MDA-MB-231 cells with or without the miR-22 inhibitor for 48 h. Cells were suspended in serum-free medium and loaded onto matrigel invasion chamber inserts. We observed that miR-22 inhibition reduces the invasive capacity of these cells (Figure 6A). To further confirm that this effect is mediated through TIP60, we transfected the stable TIP60-overexpressing MDA-MB-231 cells (lacking miR-22 binding site) with the miR-22 inhibitor, using vector-expressing cells as a control. To confirm the expression of the miR-22 and TIP60 after inhibition or overexpression of the miRNA, we quantitated the expression of miR-22 and TIP60 in the MDA-MB-231 cells transfected with or without miR-22 inhibitor and miR-22 mimic by Q-PCR (Supplementary Figure S1). We found a decrease in cell invasion in TIP60-overexpressing cells, which further confirms that the effect on cell invasion is mediated through TIP60 (Figure 6B). We also noted that, upon transfection with the miR-22 inhibitor, TIP60-overexpressing cells showed a further reduction in invasion. This may be due to an increase in endogenous TIP60 levels in these cell lines or miR-22 may target an additional factor involved in regulating invasion. Since miR-22 inhibition decreased cell invasion capacity, we next overexpressed miR-22 mimic in MCF7 cells for 48 h (Supplementary Figure S1). Cells were suspended in serum-free medium and loaded onto matrigel invasion chamber inserts. We found that miR-22 mimic overexpression increased the invasiveness of the cells (Figure 6C). These results suggest that miR-22 targets TIP60 leading to an increase in cell migration and invasion of breast cancer cells thereby promoting metastasis.

### Levels of TIP60 and miR-22 as a predictor of disease progression in breast cancer

**Figure 7:**
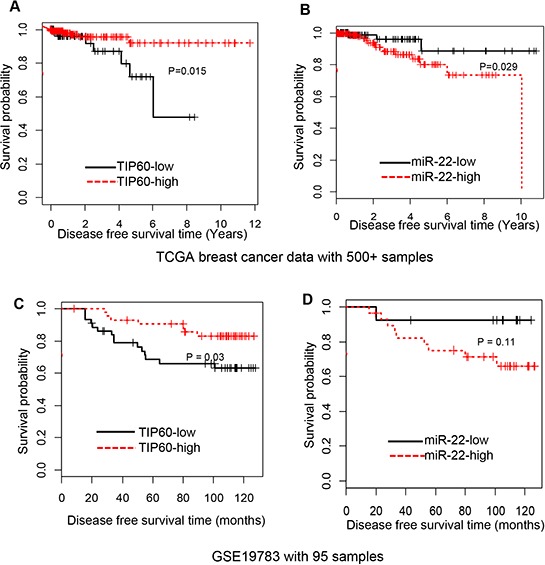
TIP60 and miR-22 expression in breast cancer tumors correlated with high and low survival, respectively **A–B.** Kaplan-Meier plot, based on breast cancer data from The Cancer Genome Atlas (TCGA), illustrates the survival probability for patients with low or high TIP60 and miR-22 expression levels in breast cancers. High expression of TIP60 leads to higher survival probability, whereas high expression of miR-22 leads to lower survival probability. *P* < 0.015 and *P* < 0.029, respectively. **C–D.** Kaplan-Meier plot, based on Gene express omnibus expression dataset (GSE19783), illustrates the survival probability for patients with low or high TIP60 and miR-22 expression levels in breast cancers. *P* < 0.03 and *P* < 0.11, respectively.

Having identified this interesting regulatory link between miR-22 and TIP60 in cell culture models and patient datasets, we sought to investigate its significance in a pathophysiological scenario. For this, we used available gene expression and survival data from the TCGA dataset and the GSE19783 from gene expression omnibus (GEO) database to compare overall survival between patient cohorts that exhibited high versus low TIP60 expression levels. We found that patients with high TIP60 and low miR-22 expression were associated with good survival prognoses (*P* = 0.015; Figure 7A, 7C) whereas patients with low TIP60 and high miR-22 levels showed poorer prognoses for survival (*P* = 0.029; Figure 7B, 7D). Breast cancer is classified into molecular subtypes; we investigated the expression of miR-22 and TIP60 in TCGA dataset for breast cancer and found no significant differences between different subtypes (Supplementary Figure S2). To rule out the possibility of other factors such as age, stage, ER status, PR status, and Her2 status determining the relationship between miR-22 and TIP60, multivariate analysis was performed. As summarized in Table 3, multivariate analysis linear regression elucidates variables significantly affecting expression level of TIP60 in breast cancer survival. Among factors such as age, stage, ER, PR and Her2 status, the strongest component that determines expression level of TIP60 in patient samples was miR-22 as illustrated by high eigenvalue (data not shown). Similarly, TIP60 was found to be the strongest factor in determining miR-22 expression level in breast cancer patient samples. Therefore, the model remained essentially unchanged when other components (age, stage, ER, PR and Her2 status) were dropped. Besides, patients with high expression of miR-22 are likely to have low expression of TIP60 and vice versa due to the negative regression coefficient of miR-22 and TIP60.

## DISCUSSION

Metastasis—a cessation of neoplastic progression—is one of the main causes of death in patients with breast cancer. Epithelial–mesenchymal transition (EMT) is thought to be one of the key processes that causes benign tumor cells to transition into invasive and metastatic cells [23]. In this study, we showed that miR-22 expression potently activates the migration and invasive capacity of basal breast cancer cells. In addition, we show evidence to suggest that miR-22 has an oncogenic function in these cells. These findings are in line with the upregulation of miR-22 in more advanced stages of breast cancer and with previous reports which have also implicated miR-22 as an oncogene in breast cancer [24, 25]. Recently, Song et al. [20] showed that miR-22 antagonizes another miRNA, miR-200, through directly targeting of the methyl cytosine dioxygenase TET (ten-11 translocation) family members and, hence, chromatin remodeling toward miR-200 transcriptional silencing. Further, Lee et al. demonstrate a central role of miR-22 in the physiological regulation of MDC1-dependent DDR, a molecular mechanism of Akt1 activation and senescence leading to increased genomic instability, which fosters an environment that promotes tumorigenesis [26]. Although these studies implicate miR-22 as an oncogene, other studies have suggested a tumor suppressor function of miR-22. miR-22 was identified as a tumor suppressor gene in human colon cancers, influencing p53-dependent cellular fate through the formation of the p53–miR-22–p21 axis [27]. Another study showed miR-22 acts as tumor suppressor by targeting the Sp1 gene and inhibiting gastric cancer cell migration and invasion [28]. Additionally, miR-22 was also implicated in activating the cellular senescence program in cancer cells and acts as a tumor suppressor [29]. A recent study also shows miR-22 acts as a tumor suppressor by targeting GLUT1 and is directly correlated with the TNM stage, local relapse, distant metastasis, and survival of breast cancer patients [30]. Further investigations along these lines will be needed to ascertain whether miR-22 is an oncogene or a tumor suppressor.

The acetyl-transferase TIP60 is a *bona fide* tumor suppressor in cancer and its expression is down-regulated in colon carcinomas [15] and lung cancers [31]. Interestingly, in colon carcinoma, the ratio between TIP60 and p400 mRNAs is important for cancer progression [32]. However, the molecular determinant and underlying mechanism is yet to be discovered. Interestingly, downregulation of TIP60 in colorectal cancer is correlated with larger tumor size, distant metastasis, and a higher stage of tumor node metastasis classification; yet, the molecular mechanism of TIP60's downregulation is not known. Our study identifies a non-coding RNA that can regulate the expression of TIP60 in breast cancer. It would be interesting to investigate whether this regulation exists in colon cancer as well.

Pathways governed by TIP60 *via* its tumor suppressor function have yet to be identified. Gorrini et al. [33] showed that TIP60 is a haplo-insufficient tumor suppressor in Eμ-myc transgenic mice, and suggested that it is required for an oncogene-induced DNA damage response. Collectively, these findings indicated that decreased TIP60 expression correlates with tumor development; but the molecular mechanism of TIP60's downregulation was still not clarified. We show, for the first time, that TIP60 is a direct target of miR-22 and its downregulation by miR-22 subsequently results in the activation of an EMT program. EMT is characterized by a loss of cell adhesion and the suppression of epithelial genes, such as *E-cadherin* concomitant with an acquisition of mesenchymal markers (including *N-cadherin, Vimentin*, and *Fibronectin*) and increased cell motility and invasiveness. Numerous miRNAs have been linked to EMT pathways. For example, miR-9 can directly regulate *E-cadherin* by targeting its 3′UTR in human mammary epithelial cells, thereby promoting mesenchymal-like characteristics of the cells with increased motility and invasiveness [34]. miR-661 is shown to regulate *Nectin-1* and *StarD10* in the disassembly of epithelial cell junctions in SNAI1-expressing breast cancer cells [35]. Recently, the expression of miR-197 was found to induce EMT along with the downregulation of p120-catenin in pancreatic cancer cells [36]. In contrast, in hepatic cancer cells, miR-194 overexpression results in reduced cell invasion, migration and metastasis by targeting *N-cadherin* [37]. Thus, we also investigated the regulatory effect of miR-22 on *E-cadherin* and *N-cadherin in vitro*. MCF7 is an estrogen alpha positive (ER+) and also an epithelial cell line. It is known that miR-22 regulates ER and miR-22 levels are reduced in ER+ cell lines [38, 39]. We showed that MCF7 cell line has reduced miR-22 levels and high TIP60 (tumor suppressor gene) levels. Overexpression of miR-22 in these cells reduced TIP60 levels and promotes invasion and migration. MDA-MB-231 being triple negative cell line (ER-, PR-, and Her2-) and a mesenchymal cell line has elevated level of miR-22 and reduce level of TIP60. We have shown that miR-22 is required to maintain the metastasis levels of the MDA-MB-231 cell line by targeting TIP60. Inhibition of miR-22 by the miR-22 inhibitor in highly metastatic MDA-MB-231 cells leads to a reduction of metastatic phenotypes, as well as an elevation of the expression of TIP60. Our data also showed that overexpression of miR-22 in MCF7 cells caused a decrease in *E-cadherin* levels and an increase in *N-cadherin* levels, thus promoting EMT. Alternatively, inhibition of miR-22 expression in MDA-MB-231 cells resulted in increased *E-cadherin* levels and suppressed EMT. Thus, miR-22 may promote EMT by inhibiting TIP60. During the initial stages of metastasis, epithelial cells undergo EMT, causing a loss of cell-cell contacts, increased motility and cell invasion. Further, in our gain- and loss-of-function experiments, we have demonstrated that miR-22 inhibition in the MDA-MB-231 metastatic cell line causes a decrease in cell migration as well as invasion, whereas its overexpression in MCF7 cells resulted in increased cell migration and invasion.

In conclusion, we have identified a novel link between miR-22 and TIP60 in breast cancer metastasis. miR-22 is upregulated in metastatic breast cancer cell lines as well as in patients with breast cancer, and causes the downregulation of TIP60 and modulation of the EMT pathway. Our study suggests that miR-22 and TIP60 levels could be used as a prognostic marker for breast cancer.

## MATERIALS AND METHODS

### Cell culture and reagents

Human breast epithelial and cancer cell lines, MCF10A (CRL-10317™), MCF-7, MDA-MB-231, were obtained from ATCC (Manassas, VA, USA). SkBR3, BT474, BT549, T47D, HS578T cells were generously provided by Prof. H. Phillip Koeffler (Cancer Science Institute, Singapore). MCF10A cells were cultured in DMEM/F12 medium supplemented with 5% horse serum and the medium was further supplemented with 20 ng/ml epithelial growth factor (EGF), 0.5 mg/ml hydrocortisone, 100 ng/ml cholera toxin, 10 μg/ml insulin. MCF-7 and MDA-MB-231 were maintained in DMEM; SkBR3 in DMEM supplemented with L-glutamine; HS578T in DMEM supplemented with insulin; and T47D and BT474 in RPMI-40 medium. All media were supplemented with 10% FBS and 100 U of penicillin and streptomycin and grown at 37°C with 5% CO_2_. All tissue culture reagents were purchased from Invitrogen (Carlsbad, CA, USA) and Sigma-Aldrich (St. Louis, MO, USA).

### Oligonucleotides, plasmids, and transfection

Lipofectamine RNAiMAX (Invitrogen) was used to transfect MCF7 and MDA-MB-231 cells. miR-22 mimic, miR mimic negative control, miR-22 inhibitor, and miR-22 negative control miRNA inhibitor were purchased from Dharmacon Research Inc. (Lafayette, CO, USA). The pmirGLO Dual-Luciferase vector was obtained from Promega (E1330; Fitchburg, WI, USA). To overexpress TIP60, the open reading frame was cloned into LPCX vector.

### Quantitative reverse transcription PCR

Total RNA from cell lines was extracted using TRIzol Reagent (Invitrogen) as per manufacturer's instructions. RNA (2 μg) was reverse transcribed in a 20-μl reaction using iScript Supermix master mix (Bio-Rad, Hercules, CA, USA). For miRNA, cDNA was synthesized using a stem-loop specific primer for miR-22, and then subjected to real time PCR using 2 μl of a 1:5 dilution of the reverse-transcribed cDNA and SYBR green in an ABI Fast Q-PCR machine (Applied Biosystems, Foster City, CA, USA). The cycling conditions were as follows: 50°C for 2 min, 95°C for 5 min, and 40 cycles of 95°C for 15 sec followed by 60°C for 1 min (annealing and extension). Primer sequences are listed in Table 1. Each reaction was performed in triplicate. The data were normalized to GAPDH and U6 expression for mRNA and miRNA, respectively. The relative expression of each gene was quantified by the ΔΔCT method.

**Table 1:** List of Q-PCR Primers

Serial No.	Name of the Gene	Forward primer Sequences	Reverse primer Sequences
1	*TIP60*	AATGTGGCCTGCATCCTAAC	TGTTTTCCCTTCCACTTTGG
2	*miR-22*	ACACTCCAGCTGGGAAGCTGCCAGTTGAAG	GGTGTCGTGGAGTCGGCAA
3	*U6*	CTCGCTTCGGCAGCACATATACT	ACGCTTCACGAATTTGCGTGTC
4	*18S*	GTAACCCGTTGAACCCCATT	CCATCCAATCGGTAGTAGCG
5	*E-cadherin*	TTACTGCCCCCAGAGGATGA	TGCAACGTCGTTACGAGTCA
6	*Epcam*	GCTGGCCGTAAACTGCTTTG	ACATTTGGCAGCCAGCTTTG
7	*N-cadherin*	CCGGTTTCATTTGAGGGCAC	TCCCTCAGGAACTGTCCCAT
8	*Fibronectin*	AACCCTTCCACACCCCAATC	ACTGGGTTGCTGACCAGAAG
9	*Snail1*	TCTTTCCTCGTCAGGAAGCC	GATCTCCGGAGGTGGGATGG
10	*Snail2*	CTCCTCATCTTTGGGGCGAG	CTTCAATGGCATGGGGGTCT
11	*Zeb1*	AGGATGACCTGCCAACAGAC	CTTCAGGCCCCAGGATTTCTT
12	miR-22 stem loop primer for c-DNA	CTCAACTGGTGTCGTGGAGTCGG CAATTCAGTTGAGACAGTTCT	

### Luciferase assays

A dual-luciferase reporter vector was used to generate the luciferase constructs. The TIP60 3′UTR, containing the predicted binding site for miR-22, was amplified from genomic DNA by PCR. The PCR product was digested by *Pme*I and *Not*I enzymes and the digested fragment was cloned into pmirGLO luciferase plasmid to obtain a wild-type luciferase construct pmirGLO-TIP60 3′-UTR. To generate point mutant and deletion constructs, the putative miR-22 binding site in TIP60 3′-UTR was mutated or deleted using Quick-change Site-Direct Mutagenesis Kit (200522–5; Stratagene, La Jolla, CA, USA) as per manufacturer's instructions. Cloning was confirmed by sequencing. Primers used for PCR and sequencing are listed in Table 2. For luciferase assays, MCF7 cells were plated in 12-well plates and 24 h later co-transfected with 50 nM miR-22 or miR mimic negative control and 50 nM miR-22 inhibitor, 100 ng pmirGLO or pmirGLO containing wild-type TIP60 3′-UTR or the corresponding mutant or deleted constructs. Forty-eight hours later, luciferase activity was measured using Dual-Luciferase Reporter Assay Kit (E1960; Promega) on a GLOmax microplate luminometer (Promega). Firefly luciferase signals were normalized using Renilla luciferase signals. All experiments were performed in triplicate.

**Table 2:** List of Cloning 3′UTR Primers

Serial No.	Name of the primers (Q-PCR)	Sequences
1	TIP60–3′UTR-Forward primer	ATATGCGGCCGCGTGACCAGACACTGCCCACT
2	TIP60–3′UTR-Reverse primer	GCGCATCGATTGCATGGCTCTGGCATATAG

**Table 3:** Multivariant analysis

Variable		Sample number	R with miR-22	*P* value
**ER-alpha_status**	positive	320	0.037	0.45
	negative	101		
**PR_status**	positive	289	0.094	0.054
	negative	132		
**Her2_status**	positive	83	0.036	0.46
	negative	338		
**Age**	< = 50	141	0.006	0.90
	> 50	280		
**Stage**	I–II	321	0.030	0.54
	III–X	100		
**TIP60**		421	0.193	< 0.0001

### Western blotting

Cells were lysed using RIPA lysis buffer [50 mM Tris (pH 7.5), 150 mM NaCl, 1% NP40, 0.25% sodium deoxycholate, 1 mM EDTA, 1 mM DTT and protease inhibitor cocktail]. Protein concentration was determined using the Bradford Protein Assay kit (500–0001; Bio-Rad). Equal amounts of protein were separated on SDS polyacrylamide gels and transferred to nitrocellulose membranes (162–0115; Bio-Rad) using the Bio-Rad semi-dry transfer apparatus. Membranes were blocked for 1 h with 5% skim milk in Tris-buffered saline containing 0.1% Tween-20, and then incubated overnight with primary antibody. Blots were then washed and incubated with secondary antibody, washed again, and visualized by chemiluminescence. β-actin (sc-81178) and α-actinin (sc-166524) were used as loading controls. The TIP60 serum antibody was generated in the lab. E-cadherin (BD-Bioscience), Vimentin (Cell Signaling), Alexa 594 secondary antibody and Alexa 594 phalloidin staining was bought from Life Technologies and mounting medium containing DAPI was purchased from Santa Cruz Biotechnology Inc.

### Immuno-fluorescence

MDA-MB-231 cells were transfected with either miR mimic negative control or miR-22 mimic. Cells were cultured on cover slips for 48 h and then immuno-fluorescence assay was performed by fixing the cells for 15 min at room temp in 3.7% paraformaldehyde. Cells were washed 3 times with PBS and permeabilized by 0.5% Triton-X-100 for 5 min. Cells were then washed 2 times with PBS and 1 time with 0.1 M glycine in PBS. Cells were incubated in E-cadherin (1:400) and Vimentin (1:100) antibodies overnight in 0.5% Triton-X-100 in PBS. Next day cells were washed 3 times with PBS and then incubated at 37°C for 30 min with secondary antibodies or with the F-actin dye. Cells were then washed 3 times with ultrapure water to remove the salts and were mounted on the slides using antifade reagent, and examined with confocal microscope (Nikon) at 60 X magnification. Similarly, MCF7 cells were transfected with either miR mimic negative control or miR-22 mimic for 48 h and immunofluorescence was performed similarly as described above.

### Wound-healing assay

MDA-MB-231, MDA-MB-231-LPCX, MDA-MB-231-LPCX-TIP60 and MCF7 cells were seeded in 12-well plates and grown to 90% confluence. Cells were transfected with or without miR-22 inhibitor or miR-22 mimic. After 36 h of transfection, cells were serum starved overnight and a linear wound was created using a pipette tip. Wound closure was monitored using live cell imaging microscopy (Nikon, Tokyo, Japan) at an interval of 30 min for 24–48 h. Wound size was then measured randomly at three sites perpendicular to the wound.

### Invasion assay

For the invasion assay, Corning BioCoat Matrigel Invasion Chambers with 8.0-μm PET Membrane were used (354480; Corning, Corning, NY, USA). As per the protocol, inserts were rehydrated for 2 h at 37°C and then MDA-MB-231, MDA-MB-231-LPCX, MDA-MB-231-LPCX-TIP60 cells transfected with or without miR-22 inhibitor and MCF7 cells transfected with or without miR-22 mimic were suspended in serum-free medium and loaded onto the chamber inserts. The inserts were placed into the wells of a 24-well plate that contained media supplemented with 10% FBS. Cells were incubated at 37°C and allowed to migrate and invade through the Matrigel and membrane pores. The upper Matrigel layer and cells were removed after 24 h (for MDA-MB-231 cells) or 72 h (for MCF7 cells) by scrubbing. The cells on the surface of the lower side of the membrane were fixed with 100% methanol and stained with Hoechst stain (33342; Life Technologies). Cells that migrated onto the lower surface were counted from representative areas using ImageJ software (NIH, Bethesda, MD).

### Stable cell lines

Virus was generated by transfecting 5 × 10^6^ 293T cells with the plasmids [MSCV construct: i.e., MSCV vector alone (MSCV) and TIP60 overexpressing vector (MSCV TIP60) and LPCX construct: i.e., LPCX vector alone (LPCX) and TIP60 overexpressing vector (LPCX TIP60)], using Lipofectamine 2000, as per manufacturer's protocol. Viruses were harvested after 72 h of transfection and were used to infect 1 × 10^6^ MCF7 or 2 × 10^6^ MDA-MB-231-luc-D3H2LN cells together with polybrene (107689, Sigma-Aldrich) reagent (0.4 mg/ml). After 6 h, media containing the virus was replaced by growth media. After 24 h, puromycin was added into the growth media for selection. Media with antibiotics was changed every 48 h until the mock-transfected cells died. The cells were continuously selected for 2 weeks for the generation of stable cell lines.

### Bioinformatics analysis

For survival data analysis, raw gene expression data were downloaded from The Cancer Genome Atlas (TCGA) breast cancer database (https://tcga-data.nci.nih.gov/tcga/) and from GEO databases, respectively. The downloaded TCGA breast cancer data were the RNA-seq dataset of level 3 and normalization of the data was performed based on the total mapable reads. For microarray data (GSE19783), the Cross-Correlation method was used for data normalization [40]. In the survival analysis, the median intensity cross all samples was first used to classify the samples into the respective expression high and low groups. In order to minimize the false positives in classification of high and low expression groups, the samples with middle expression within the 15% range from the median expression value were removed. The analysis of the survival data was based on the Kaplan-Meier method. Gene-Set Enrichment Analysis (GSEA) was performed using msigdb.v4 (http://www.broad.mit.edu/gsea/), comparing 28 TCGA samples with high TIP60/low miR-22 expression versus 28 samples with low TIP60/high miR-22 expression.

A correlation of TIP60 with miR-22 was obtained based on the normalized data cross all samples in the cohort. In order to ensure that this correlation is not independent of subtypes, the multivariate analysis was performed. Using 400+ TCGA breast cancer samples available with all these factors, we performed principal component analysis (PCA) to identify the contributing fraction of each principal component (PC), and found that the first PC is dominant and contributes 98.2% among all PCs. Multiple linear regression with miR-22 as the dependent variable was performed, and the exploratory variables for the multiple regression included not only TIP60 expression but also the age, stage, ER status, PR status, and Her2 status.

## SUPPLEMENTARY FIGURES



## References

[1] Wang Q, Zhao ZB, Wang G, Hui Z, Wang MH, Pan JF, Zheng H (2013). High expression of KIF26B in breast cancer associates with poor prognosis. PloS one.

[2] Bartel DP (2004). MicroRNAs: genomics, biogenesis, mechanism, and function. Cell.

[3] Lee RC, Feinbaum RL, Ambros V (1993). The C. elegans heterochronic gene lin-4 encodes small RNAs with antisense complementarity to lin-14. Cell.

[4] Calin GA, Liu CG, Sevignani C, Ferracin M, Felli N, Dumitru CD, Shimizu M, Cimmino A, Zupo S, Dono M, Dell'Aquila ML, Alder H, Rassenti L, Kipps TJ, Bullrich F, Negrini M (2004). MicroRNA profiling reveals distinct signatures in B cell chronic lymphocytic leukemias. Proceedings of the National Academy of Sciences of the United States of America.

[5] Iorio MV, Ferracin M, Liu CG, Veronese A, Spizzo R, Sabbioni S, Magri E, Pedriali M, Fabbri M, Campiglio M, Menard S, Palazzo JP, Rosenberg A, Musiani P, Volinia S, Nenci I (2005). MicroRNA gene expression deregulation in human breast cancer. Cancer research.

[6] Lu J, Getz G, Miska EA, Alvarez-Saavedra E, Lamb J, Peck D, Sweet-Cordero A, Ebert BL, Mak RH, Ferrando AA, Downing JR, Jacks T, Horvitz HR, Golub TR (2005). MicroRNA expression profiles classify human cancers. Nature.

[7] Michael MZ, SM OC, van Holst Pellekaan NG, Young GP, James RJ (2003). Reduced accumulation of specific microRNAs in colorectal neoplasia. Molecular cancer research: MCR.

[8] Volinia S, Calin GA, Liu CG, Ambs S, Cimmino A, Petrocca F, Visone R, Iorio M, Roldo C, Ferracin M, Prueitt RL, Yanaihara N, Lanza G, Scarpa A, Vecchione A, Negrini M (2006). A microRNA expression signature of human solid tumors defines cancer gene targets. Proceedings of the National Academy of Sciences of the United States of America.

[9] Cui J, Bi M, Overstreet AM, Yang Y, Li H, Leng Y, Qian K, Huang Q, Zhang C, Lu Z, Chen J, Sun T, Wu R, Sun Y, Song H, Wei X (2015). MiR-873 regulates ERalpha transcriptional activity and tamoxifen resistance via targeting CDK3 in breast cancer cells. Oncogene.

[10] Ujihira T, Ikeda K, Suzuki T, Yamaga R, Sato W, Horie-Inoue K, Shigekawa T, Osaki A, Saeki T, Okamoto K, Takeda S, Inoue S (2015). MicroRNA-574–3p, identified by microRNA library-based functional screening, modulates tamoxifen response in breast cancer. Scientific reports.

[11] Ikura T, Ogryzko VV, Grigoriev M, Groisman R, Wang J, Horikoshi M, Scully R, Qin J, Nakatani Y (2000). Involvement of the TIP60 histone acetylase complex in DNA repair and apoptosis. Cell.

[12] Kamine J, Elangovan B, Subramanian T, Coleman D, Chinnadurai G (1996). Identification of a cellular protein that specifically interacts with the essential cysteine region of the HIV-1 Tat transactivator. Virology.

[13] Berns K, Hijmans EM, Mullenders J, Brummelkamp TR, Velds A, Heimerikx M, Kerkhoven RM, Madiredjo M, Nijkamp W, Weigelt B, Agami R, Ge W, Cavet G, Linsley PS, Beijersbergen RL, Bernards R (2004). A large-scale RNAi screen in human cells identifies new components of the p53 pathway. Nature.

[14] Chen G, Cheng Y, Tang Y, Martinka M, Li G (2012). Role of Tip60 in human melanoma cell migration, metastasis, and patient survival. J Invest Dermatol.

[15] Sakuraba K, Yasuda T, Sakata M, Kitamura YH, Shirahata A, Goto T, Mizukami H, Saito M, Ishibashi K, Kigawa G, Nemoto H, Sanada Y, Hibi K (2009). Down-regulation of Tip60 gene as a potential marker for the malignancy of colorectal cancer. Anticancer research.

[16] Gupta A, Jha S, Engel DA, Ornelles DA, Dutta A (2013). Tip60 degradation by adenovirus relieves transcriptional repression of viral transcriptional activator EIA. Oncogene.

[17] Jha S, Vande Pol S, Banerjee NS, Dutta AB, Chow LT, Dutta A (2010). Destabilization of TIP60 by human papillomavirus E6 results in attenuation of TIP60-dependent transcriptional regulation and apoptotic pathway. Molecular cell.

[18] Reitsma JM, Savaryn JP, Faust K, Sato H, Halligan BD, Terhune SS (2011). Antiviral inhibition targeting the HCMV kinase pUL97 requires pUL27-dependent degradation of Tip60 acetyltransferase and cell-cycle arrest. Cell host & microbe.

[19] Subbaiah VK, Zhang Y, Rajagopalan D, Abdullah LN, Yeo-Teh NS, Tomaic V, Banks L, Myers MP, Chow EK, Jha S (2015). E3 ligase EDD1/UBR5 is utilized by the HPV E6 oncogene to destabilize tumor suppressor TIP60. Oncogene.

[20] Song SJ, Poliseno L, Song MS, Ala U, Webster K, Ng C, Beringer G, Brikbak NJ, Yuan X, Cantley LC, Richardson AL, Pandolfi PP (2013). MicroRNA-antagonism regulates breast cancer stemness and metastasis via TET-family-dependent chromatin remodeling. Cell.

[21] Tan TZ, Miow QH, Miki Y, Noda T, Mori S, Huang RY, Thiery JP (2014). Epithelial-mesenchymal transition spectrum quantification and its efficacy in deciphering survival and drug responses of cancer patients. EMBO molecular medicine.

[22] Kalluri R, Weinberg RA (2009). The basics of epithelial-mesenchymal transition. J Clin Invest.

[23] Wang L, Wang J (2012). MicroRNA-mediated breast cancer metastasis: from primary site to distant organs. Oncogene.

[24] Song SJ, Ito K, Ala U, Kats L, Webster K, Sun SM, Jongen-Lavrencic M, Manova-Todorova K, Teruya-Feldstein J, Avigan DE, Delwel R, Pandolfi PP (2013). The oncogenic microRNA miR-22 targets the TET2 tumor suppressor to promote hematopoietic stem cell self-renewal and transformation. Cell stem cell.

[25] Wang X, Wang HK, Li Y, Hafner M, Banerjee NS, Tang S, Briskin D, Meyers C, Chow LT, Xie X, Tuschl T, Zheng ZM (2014). microRNAs are biomarkers of oncogenic human papillomavirus infections. Proceedings of the National Academy of Sciences of the United States of America.

[26] Lee JH, Park SJ, Jeong SY, Kim MJ, Jun S, Lee HS, Chang IY, Lim SC, Yoon SP, Yong J, You HJ (2015). MicroRNA-22 Suppresses DNA Repair and Promotes Genomic Instability through Targeting of MDC1. Cancer research.

[27] Tsuchiya N, Izumiya M, Ogata-Kawata H, Okamoto K, Fujiwara Y, Nakai M, Okabe A, Schetter AJ, Bowman ED, Midorikawa Y, Sugiyama Y, Aburatani H, Harris CC, Nakagama H (2011). Tumor suppressor miR-22 determines p53-dependent cellular fate through post-transcriptional regulation of p21. Cancer research.

[28] Guo MM, Hu LH, Wang YQ, Chen P, Huang JG, Lu N, He JH, Liao CG (2013). miR-22 is down-regulated in gastric cancer, and its overexpression inhibits cell migration and invasion via targeting transcription factor Sp1. Medical oncology.

[29] Xu D, Takeshita F, Hino Y, Fukunaga S, Kudo Y, Tamaki A, Matsunaga J, Takahashi RU, Takata T, Shimamoto A, Ochiya T, Tahara H (2011). miR-22 represses cancer progression by inducing cellular senescence. J Cell Biol.

[30] Chen B, Tang H, Liu X, Liu P, Yang L, Xie X, Ye F, Song C, Xie X, Wei W (2015). miR-22 as a prognostic factor targets glucose transporter protein type 1 in breast cancer. Cancer Lett.

[31] Lleonart M, Vidal F, Gallardo D, Diaz-Fuertes M, Rojo F, Cuatrecasas M, Lopez-Vicente L, Kondoh H, Blanco C, Carnero A, Ramon y Cajal S (2006). New p53 related genes in human tumors: significant downregulation in colon and lung carcinomas. Oncology reports.

[32] Mattera L, Escaffit F, Pillaire MJ, Selves J, Tyteca S, Hoffmann JS, Gourraud PA, Chevillard-Briet M, Cazaux C, Trouche D (2009). The p400/Tip60 ratio is critical for colorectal cancer cell proliferation through DNA damage response pathways. Oncogene.

[33] Gorrini C, Squatrito M, Luise C, Syed N, Perna D, Wark L, Martinato F, Sardella D, Verrecchia A, Bennett S, Confalonieri S, Cesaroni M, Marchesi F, Gasco M, Scanziani E, Capra M (2007). Tip60 is a haplo-insufficient tumour suppressor required for an oncogene-induced DNA damage response. Nature.

[34] Ma L, Young J, Prabhala H, Pan E, Mestdagh P, Muth D, Teruya-Feldstein J, Reinhardt F, Onder TT, Valastyan S, Westermann F, Speleman F, Vandesompele J, Weinberg RA (2010). miR-9, a MYC/MYCN-activated microRNA, regulates E-cadherin and cancer metastasis. Nature cell biology.

[35] Vetter G, Saumet A, Moes M, Vallar L, Le Bechec A, Laurini C, Sabbah M, Arar K, Theillet C, Lecellier CH, Friederich E (2010). miR-661 expression in SNAI1-induced epithelial to mesenchymal transition contributes to breast cancer cell invasion by targeting Nectin-1 and StarD10 messengers. Oncogene.

[36] Hamada S, Satoh K, Miura S, Hirota M, Kanno A, Masamune A, Kikuta K, Kume K, Unno J, Egawa S, Motoi F, Unno M, Shimosegawa T (2013). miR-197 induces epithelial-mesenchymal transition in pancreatic cancer cells by targeting p120 catenin. Journal of cellular physiology.

[37] Meng Z, Fu X, Chen X, Zeng S, Tian Y, Jove R, Xu R, Huang W (2010). miR-194 is a marker of hepatic epithelial cells and suppresses metastasis of liver cancer cells in mice. Hepatology.

[38] Xiong J, Yu D, Wei N, Fu H, Cai T, Huang Y, Wu C, Zheng X, Du Q, Lin D, Liang Z (2010). An estrogen receptor alpha suppressor, microRNA-22, is downregulated in estrogen receptor alpha-positive human breast cancer cell lines and clinical samples. FEBS J.

[39] Pandey DP, Picard D (2009). miR-22 inhibits estrogen signaling by directly targeting the estrogen receptor alpha mRNA. Molecular and cellular biology.

[40] Chua SW, Vijayakumar P, Nissom PM, Yam CY, Wong VV, Yang H (2006). A novel normalization method for effective removal of systematic variation in microarray data. Nucleic acids research.

