# Supplementary material for: TIP60-miR-22 axis as a prognostic marker of breast cancer progression
Source: Oncotarget. 2015 Oct 19;6(38):41290–306. doi: 10.18632/oncotarget.5636 (PMC4747406; doi:10.18632/oncotarget.5636)
Supplement: Supplementary file 1 [file oncotarget-06-41290-s001.pdf]

SUPPLEMENTARY FIGURES

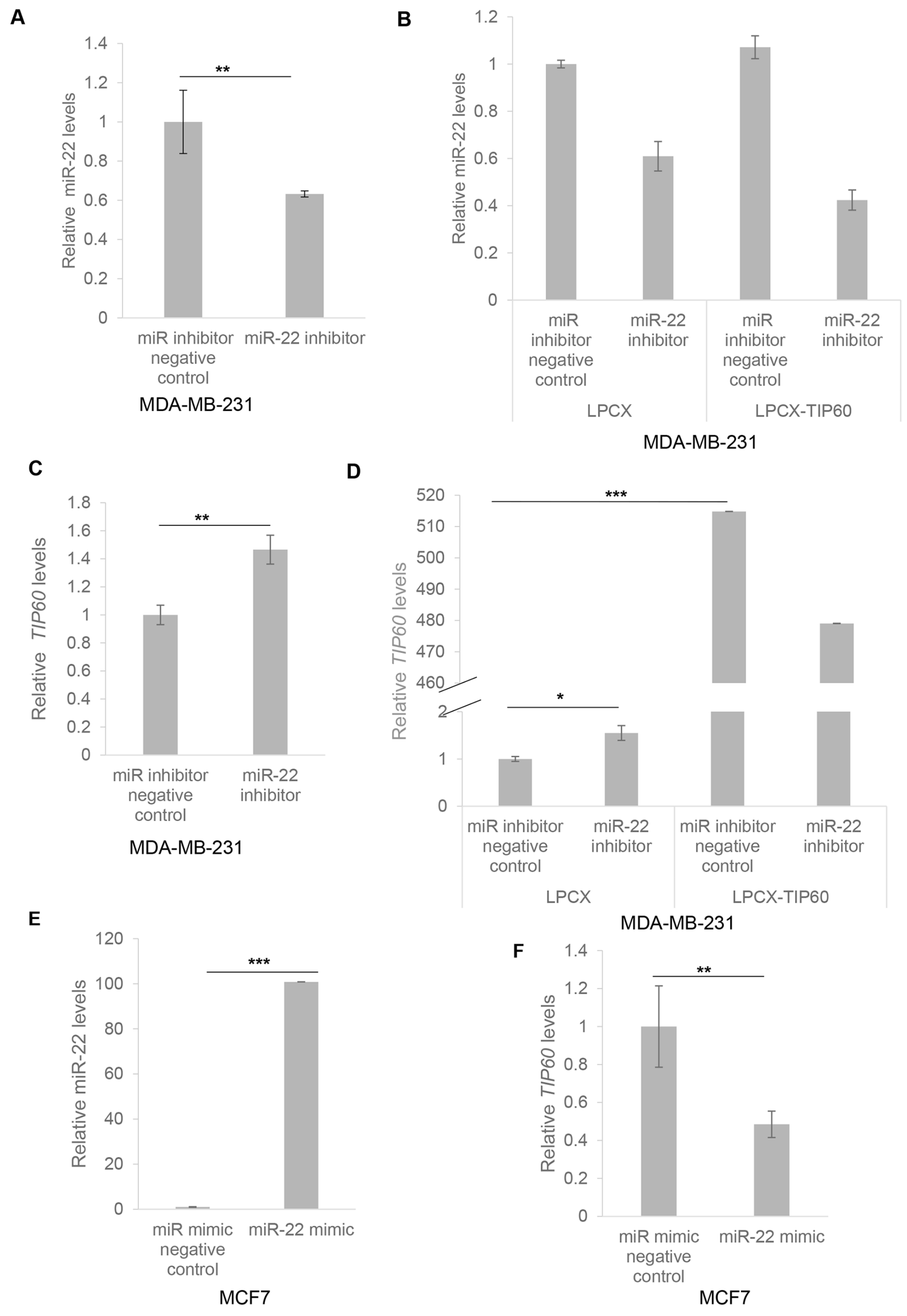

Supplementary Figure S1: TIP60 and miR-22 mRNA levels in MDA-MB-231 and MCF7 cells. A. QRT-PCR was performed to detect the levels of miR-22 on miR-22 inhibition (50 nM) in MDA-MB-231. **B.** QRT-PCR was performed to detect the levels of miR-22 on miR-22 inhibition in MDA-MB-231-LPCX and MDA-MB-231-LPCX-TIP60 cells transfected with miR-22 inhibitor for 48 h (50 nM). **C.** QRT-PCR was performed to detect the mRNA level of *TIP60* following inhibition of miR-22 in MDA-MB-231. **D.** QRT-PCR was performed to detect the mRNA level of *TIP60* in MDA-MB-231-LPCX and MDA-MB-231-LPCX-TIP60 stable cells. **E.** Relative miR-22 levels determined by QRT-PCR on miR-22 mimic overexpression (miR-22 OE, 50 nM) in MCF7 cells. **F.** mRNA expression of *TIP60* following miR-22 overexpression (miR-22 OE, 50 nM) in MCF7 cells. Data represented is compiled from three independent experiments performed in triplicate. Significance is represented as *$P < 0.05$; **$P < 0.01$; ***$P < 0.001$.

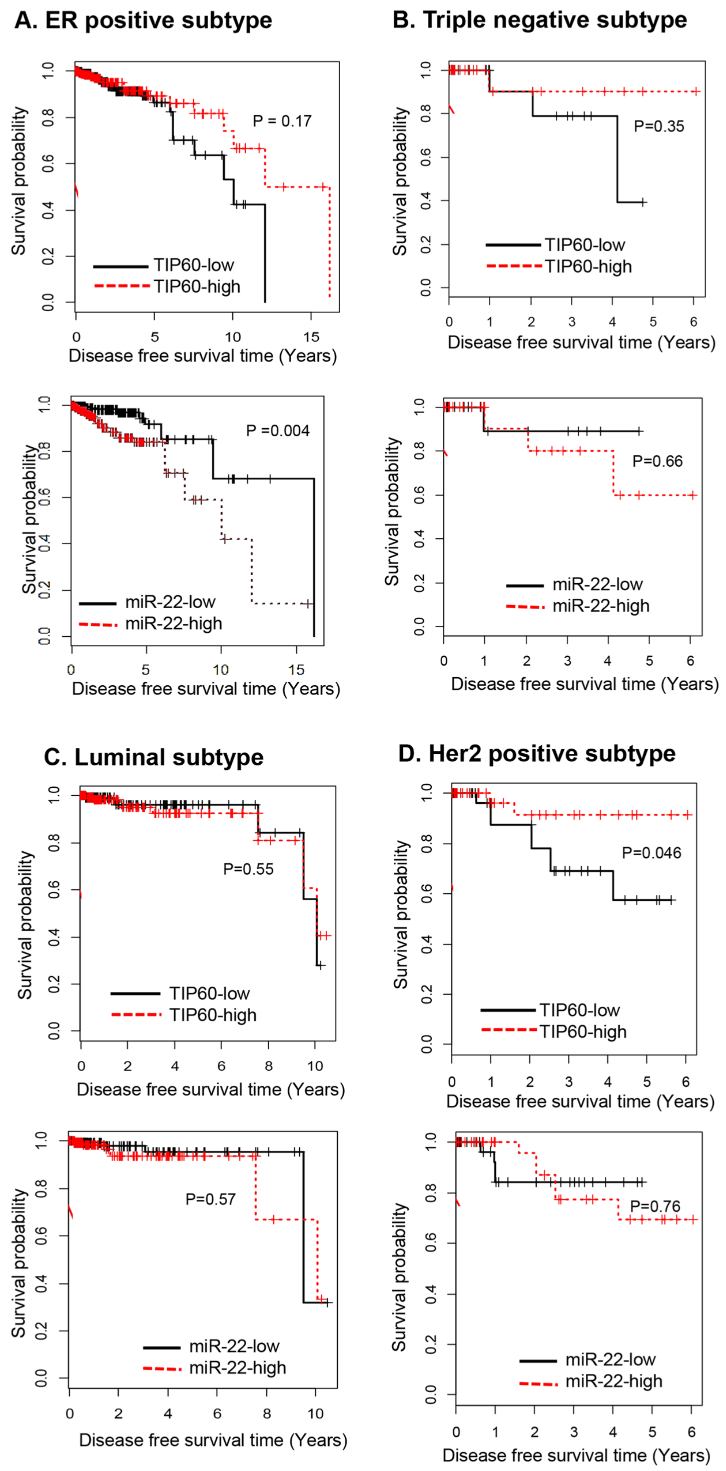

Supplementary Figure S2: TIP60 and miR-22 expression in breast cancer tumors categorized in different subtypes, correlated with high and low survival, respectively. A. Kaplan–Meier plot, based on breast cancer data from The Cancer Genome Atlas (TCGA), illustrates the survival probability for patients with low or high TIP60 and miR-22 expression levels in ER+ subtype. $P < 0.17$ and $P < 0.004$, respectively. **B.** Kaplan-Meier plot, based on breast cancer data from The Cancer Genome Atlas (TCGA), illustrates the survival probability for patients with low or high TIP60 and miR-22 expression levels in Triple negative breast cancer subtype. $P < 0.35$ and $P < 0.66$, respectively. **C.** Kaplan-Meier plot, based on breast cancer data from The Cancer Genome Atlas (TCGA), illustrates the survival probability for patients with low or high TIP60 and miR-22 expression levels in Luminal breast cancer subtype. $P < 0.55$ and $P < 0.57$, respectively. **D.** Kaplan-Meier plot, based on breast cancer data from The Cancer Genome Atlas (TCGA), illustrates the survival probability for patients with low or high TIP60 and miR-22 expression levels in Her2 positive breast cancer subtype. $P < 0.046$ and $P < 0.76$, respectively.
